# IL-25 and IL-33 Contribute to Development of Eosinophilic Airway Inflammation in Epicutaneously Antigen-Sensitized Mice

**DOI:** 10.1371/journal.pone.0134226

**Published:** 2015-07-31

**Authors:** Hideaki Morita, Ken Arae, Hirotoshi Unno, Sumika Toyama, Kenichiro Motomura, Akio Matsuda, Hajime Suto, Ko Okumura, Katsuko Sudo, Takao Takahashi, Hirohisa Saito, Kenji Matsumoto, Susumu Nakae

**Affiliations:** 1 Department of Pediatrics, Keio University School of Medicine, Tokyo, 160–8582, Japan; 2 Department of Allergy and Clinical Immunology, National Research Institute for Child Health and Development, Tokyo 157–8535, Japan; 3 Department of Pediatrics, Jikei University School of Medicine, Tokyo 105–8461, Japan; 4 Department of Immunology, Faculty of Health Science, Kyorin University, Tokyo, 192–8508, Japan; 5 Atopy Research Center, Juntendo University School of Medicine, Tokyo, 113–8412, Japan; 6 Animal Research Center, Tokyo Medical University, Tokyo 160–8402, Japan; 7 Laboratory of Systems Biology, Center for Experimental Medicine and Systems Biology, The Institute of Medical Science, The University of Tokyo, Tokyo, 108–8639, Japan; 8 Precursory Research for Embryonic Science and Technology (PRESTO), Japan Science and Technology Agency, Saitama 332–0012, Japan; French National Centre for Scientific Research, FRANCE

## Abstract

**Background:**

IL-25, IL-33 and TSLP are produced predominantly by epithelial cells and are known to induce Th2-type cytokines. Th2-type cytokines are involved not only in host defense against nematodes, but also in the development of Th2-type allergic diseases. TSLP was reported to be crucial for development of allergic airway inflammation in mice after inhalation of allergens to which they had been sensitized epicutaneously (EC) beforehand. However, the roles of IL-25 and IL-33 in the setting remain unclear.

**Methods:**

Mice deficient in IL-25 and IL-33 were sensitized EC with ovalbumin (OVA) and then challenged intranasally with OVA. Airway inflammation, the number of inflammatory cells in bronchoalveolar lavage fluids (BALFs) and airway hyperresponsiveness (AHR) in the mice were determined, respectively, by histological analysis, with a hemocytometer, and by using plethysmograph chambers with a ventilator. Expression of mRNA in the skin and lungs was determined by quantitative PCR, while the BALF levels of myeloperoxidase (MPO) and eosinophil peroxidase (EPO) and the serum levels of IgE were determined by ELISA.

**Results:**

**N**ormal OVA-specific Th2- and Th17-cell responses of lymph nodes and spleens were observed in IL-25-deficient (IL-25^-/-^) and IL-33^-/-^ mice after EC sensitization with OVA. Nevertheless, the number of eosinophils, but not neutrophils, in the BALFs, and the levels of Th2 cytokines, but not Th17 cytokines, in the lungs were significantly decreased in the IL-25^-/-^ and IL-33^-/-^ mice pre-sensitized EC with OVA, followed by inhalation of OVA, whereas their levels of AHR and OVA-specific serum IgE were normal.

**Conclusions:**

Both IL-25 and IL-33 are critical for induction of Th2-type cytokine-mediated allergic airway eosinophilia, but not Th17-type cytokine-mediated airway neutrophilia, at the local sites of lungs in the challenge phase of mice sensitized EC with OVA. They do not affect OVA-specific T-cell induction in the sensitization phase.

## Introduction

Sensitization with allergens via the upper and lower respiratory tracts due to dysfunction and/or disruption of epithelial barriers is considered to be a major route of development of asthma [1]. Transdermal allergen sensitization due to dysfunction and/or disruption of epidermal barriers is recently recognized to be another route [2–4]. In support of this, in spite of the fact that filaggrin, which is known to be crucial for formation of epidermal barriers, is expressed in the skin but not in the lung, genetic deficiency of *filaggrin* resulted in increased susceptibility to asthma as well as peanut allergy [5]. Thus, filaggrin mutations may be a predisposing factor for such diseases [5]. However, the molecular mechanisms of the transdermal allergen sensitization pathway are not fully understood.

IL-25 (an IL-17 cytokine family member), IL-33 (an IL-1 cytokine family member) and thymic stromal lymphopoietin (TSLP; an IL-7 cytokine family member) are produced predominantly by epithelial cells such as airway epithelial cells and keratinocytes. These cytokines can induce production of Th2-type cytokines such as IL-4, IL-5 and/or IL-13 by various types of cells, including Th2 cells, NKT cells, mast cells, basophils, eosinophils and type 2 innate lymphoid cells (ILC2) [6–8]. IL-25, IL-33 and/or TSLP were increased in specimens from patients with asthma [9–11] and in inflamed skin lesions of patients with atopic dermatitis [12–15]. Therefore, these cytokines may be produced by epithelial cells after exposure to allergens, contributing to the development of allergic diseases by inducing early immune responses leading to sensitization to allergens. That is, keratinocyte-derived IL-25, IL-33 and/or TSLP may be involved in sensitization to allergens, contributing to the development of not only atopic dermatitis but also asthma. Indeed, skin-derived TSLP also contributed to the development of allergic airway inflammation in mice [16]. In addition, we previously demonstrated that ST2, which is a component of IL-33R, was important for Th2-type airway inflammation in epicutaneously (EC) antigen-sensitized ST2^-/-^ mice [17]. On the other hand, it was reported that the phenotypes of ST2^-/-^ mice did not completely correspond with those of IL-33^-/-^ mice in certain disease models, such as OVA-induced airway inflammation and collagen-induced arthritis. For example, OVA-induced airway inflammation developed normally in ST2^-/-^ mice [10], but was attenuated in IL-33^-/-^ mice [18, 19]. Conversely, collagen-induced arthritis was attenuated in ST2^-/-^ mice [20], but developed normally in IL-33^-/-^ mice [21]. It was also reported that full-length IL-33 can induce inflammation in an ST2-independent fashion [22]. These observations suggest that IL-33^-/-^ mice, rather than ST2^-/-^ mice, should be used to elucidate the role of IL-33 in development of Th2-type airway inflammation in EC antigen-sensitized mice.

IL-25 is known to be responsible for development of Th2-type allergic airway inflammation after intranasal OVA challenge of mice sensitized with OVA intraperitoneally [23], but it is not essential for that reaction in mice after intranasal house dust mite extract challenge [24]. Those findings suggest that the requirement for IL-25 in development of allergic airway inflammation differs among distinct experimental models of allergic airway inflammation, at least in mice. On the other hand, we remain in the dark regarding the roles of IL-25 as well as IL-33 in allergic airway inflammation in individuals who had been epicutaneously sensitized to certain antigens. Therefore, in the present study, we investigated the roles of IL-25 and IL-33 in the development of allergic airway inflammation in epicutaneously antigen-sensitized IL-25^-/-^ mice and IL-33^-/-^ mice.

## Materials and Methods

### Ethics Statement

All procedures were approved by the Institutional Review Board of National Research Institute for Child Health and Development (Approval No. A2012-004-C02) and The Institute of Medical Science, The University of Tokyo (Approval No. A11-18). All treatments were performed under isoflurane anesthesia, and all efforts were made to minimize suffering. After experiments, mice were sacrificed by cervical spine fracture dislocation.

### Mice

BALB/cA wild-type (WT) mice were purchased from SLC, Japan (Shizuoka, Japan). Chimeric mice generated using IL-25-gene-targeted 129 ES cells were obtained from Lexicon Pharmaceuticals, Inc. (The Woodlands, TX). IL-25^-/-^ mice and IL-33^-/-^ mice on the BALB/c background were generated as described elsewhere [18, 25]. Six- to 10-week-old female mice were used in all experiments. Beginning from at least 2 weeks before starting the experiments, mice were co-housed under specific-pathogen-free conditions at the National Research Institute for Child Health and Development and The Institute of Medical Science, The University of Tokyo.

### Epicutaneous (EC) sensitization

Mice were epicutaneously (EC) sensitized with OVA, as described elsewhere [17]. In brief, the dorsal skin of mice was shaved and then stripped 6 times with adhesive cellophane tape (Nichiban Co., Ltd., Tokyo). A Finn chamber (ϕ8 mm; Smart Practice, Phoenix, AZ, U.S.A.) containing 400 μg of OVA (grade V; Sigma-Aldrich, St. Louis, MO, U.S.A.) in 40 μl of PBS or PBS alone was applied to the tape-stripped skin lesions for 3 days and then removed. One week later, a fresh patch having the same content was applied to the same skin site. This cycle was repeated 3 times, resulting in a total of 9 days’ exposure to OVA for each mouse.

### OVA-induced airway inflammation

One week after removal of the last patch, the mice were intranasally challenged with OVA (200 μg in 20 μl of PBS) or PBS alone, on 3 consecutive days. Twenty-four hours after the last challenge, bronchoalveolar lavage fluids (BALFs) were collected, and the total cell count and leukocyte profile were determined with a hemocytometer (Sysmex XT-1800i; Sysmex Corporation, Hyogo, Japan), as described previously [18].

### Measurement of myeloperoxidase (MPO) and eosinophil peroxidase (EPO) activities

MPO and EPO activities in BALFs were measured as described elsewhere [26]. Recombinant human MPO and EPO, as standard reagents, were obtained from Calbiochem.

### Quantitative real-time PCR

At 24 hours after the last intranasal challenge with OVA or PBS, the lungs were harvested and homogenized. The total RNA in the lung homogenates was isolated, and the mRNA expression levels were determined by quantitative real-time PCR, as described elsewhere [17]. To determine the exact copy numbers of the target genes, quantified concentrations of the purified PCR products of each gene were serially diluted and used as standards. Aliquots of cDNA equivalent to 5 ng of the total RNA samples were used for each qPCR. The mRNA expression levels were normalized to the GAPDH level in each sample.

### Histology

At 24 hours after the last intranasal challenge with OVA or PBS, the lungs were harvested and fixed in Carnoy’s solution. Each fixed tissue was embedded in paraffin and sliced into 5-μm sections, followed by hematoxylin-eosin staining and periodic acid-Schiff staining (PAS).

### Measurement of OVA-specific cytokine production *in vitro*

Twenty-four hours after the last EC sensitization with OVA, the spleen and draining lymph nodes (DLN), such as the axillary and inguinal lymph nodes, were harvested. Single-cell suspensions of spleen and DLN cells at 2 x 10^6^/ml in RPMI1640 (Gibco) containing 10% FCS, 0.05 mM 2-mercaptoethanol, 100 U/ml penicillin and 100 mg/ml streptomycin were cultured in 96-well plates in the presence and absence of 200 μg/ml OVA. Culture supernatants were harvested after 96-hour incubation. The levels of cytokines in the culture supernatants were determined using mouse IL-4, IL-5, IL-13 and IL-17A ELISA kits (eBioscience) according to the manufacturer’s instructions.

### Measurement of OVA-specific immunoglobulins in sera

Sera were collected at 24 hours after the last intranasal challenge with OVA or PBS. The serum levels of OVA-specific IgE were determined by ELISA, as described elsewhere [27].

### Measurement of airway hyperresponsiveness (AHR)

At 24 hours after the last intranasal challenge with OVA or PBS, AHR to methacholine (SIGMA-Aldrich) was measured as described elsewhere [28]. Briefly, mice were deeply anesthetized with ketamine (100 mg/kg, i.p.; DAIICHI SANKYO Company; Tokyo, Japan) and xylazine (10 mg/kg, i.p.; SIGMA-Aldrich), and the airway was surgically intubated. The intubated mice were connected to plethysmograph chambers with a ventilator (Elan Series Mouse RC Site; Buxco Electronics, Sharon, CT, U.S.A.) and mechanically ventilated at 150 breaths/min and a tidal volume of 0.2 ml. Aerosolized methacholine (10 ml) was administered for 10 seconds at a tidal volume of 0.2 ml. For as many as 3 times after each aerosol challenge, the data for R_L_ were continuously monitored using BioSystem XA software (Buxco Electronics).

### Statistics

Unless otherwise specified, ANOVA (measurement of AHR) and the unpaired Student’s *t*-test, two-tailed, were used for statistical evaluation of the results. All results are shown as the mean + SEM.

## Results

### IL-25 and IL-33 are not essential for antigen sensitization through tape-stripped skin lesions

**Fig 1:**
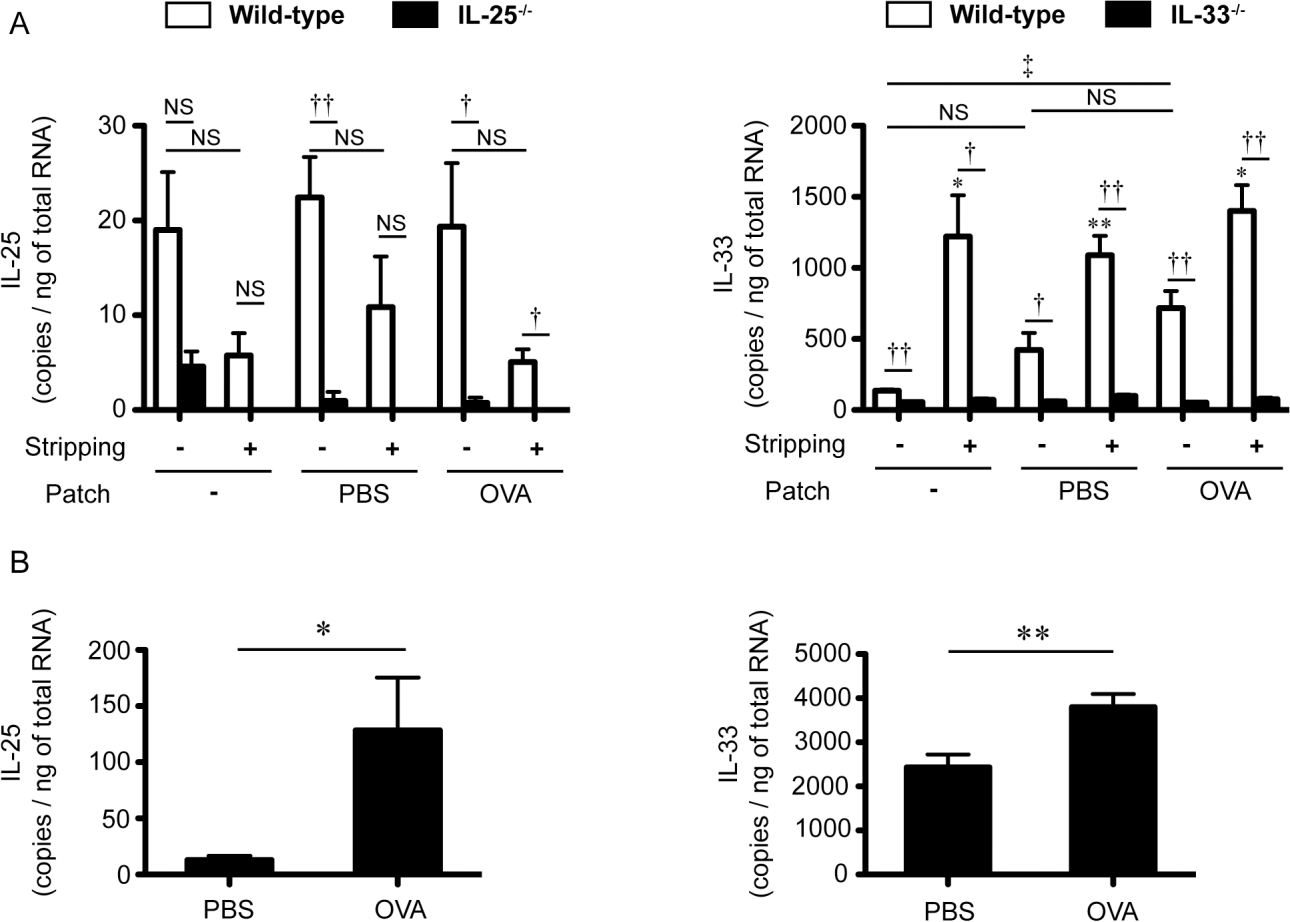
Expression of IL-25 and IL-33 mRNA in the skin after tape-stripping and in the lungs after antigen challenge. Dorsal skin was collected from wild-type (WT) mice, IL-25^-/-^ mice and IL-33^-/-^ mice 6 hours after tape-stripping followed by with or without a patch containing PBS or OVA, while lungs were harvested from WT mice sensitized EC with OVA 24 hours after the last intranasal challenge with OVA. mRNA was isolated from the skin and lungs, and the expression levels of IL-25 and IL-33 mRNA were determined by quantitative PCR. (A) The IL-25 and IL-33 mRNA expression levels in the skin (n = 3–5). *P < 0.05 vs. the corresponding values for a group without stripping (a group without stripping vs. a group with tape-stripping). ‡ P<0.05 vs. the indicated group (a group without stripping and patch vs. a group without stripping and with patch containing OVA). †*P* < 0.05 and ††*P* < 0.01 vs. the indicated group (WT vs. IL-25^-/-^, or WT vs. IL-33^-/-^). NS means “not significant” between the indicated groups. (B) The IL-25 and IL-33 mRNA expression levels in the lungs (PBS, n = 8; OVA, n = 24). Data show the mean + SEM. *P < 0.05 and **P < 0.01 (PBS group vs. OVA group).

**Fig 2:**
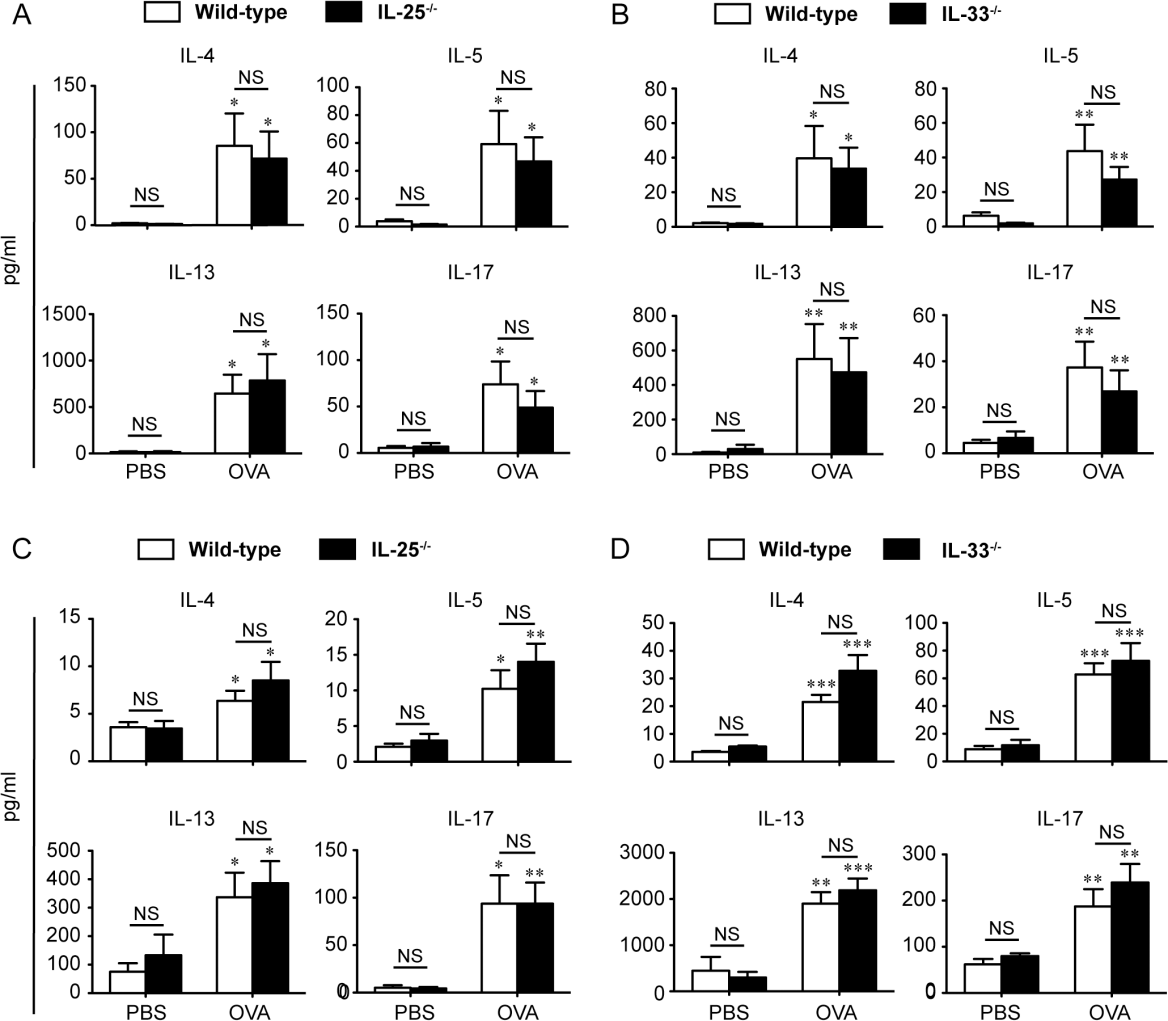
Normal Th2- and Th17-type cytokine production by DLN and spleen cells of IL-25^-/-^ and IL-33^-/-^ mice sensitized epicutaneously (EC) with OVA. DLN and spleens were collected from mice 24 hours after the last EC sensitization with OVA. DLN and spleen cells were cultured with OVA in PBS or PBS alone. ELISA was performed to determine the levels of IL-4, IL-5, IL-13 and IL-17 in the culture supernatants of the DLN cells from wild-type (WT) mice and IL-25^-/-^ mice (A) and from WT mice and IL-33^-/-^ mice (B), and the spleen cells from WT mice and IL-25^-/-^ mice (C) and from WT mice and IL-33^-/-^ mice (D). Data show the mean + SEM (n = 8–23). *P < 0.05, **P < 0.01 and ***P < 0.001 vs. the corresponding values for the culture with PBS alone. NS means “not significant” between the indicated group (WT vs. IL-25^-/-^, or WT vs. IL-33^-/-^).

We found constitutive expression of IL-25 and IL-33 mRNAs in untreated (no stripping or patch) intact skin (Fig 1A and S1 Table). Expression of IL-25 mRNA seemed to be similarly, but not significantly, reduced, in tape-stripped skin, regardless of the presence or absence of patches containing of OVA or PBS (Fig 1A and S1 Table). Expression of IL-33 mRNA was increased in the local skin of mice after tape-stripping, irrespective of the presence or absence of patches containing of OVA or PBS, compared with intact skin (Fig 1A and S1 Table). Expression of IL-25 and IL-33 mRNAs was increased in the lungs after OVA challenges (Fig 1B and S2 Table). Such changes in the expression levels of IL-25 and IL-33 mRNAs suggest that both IL-25 and IL-33 may contribute to development of airway inflammation in mice sensitized EC with OVA after tape-stripping. IL-25 was reported to enhance differentiation of Th2 cells from naïve CD4^+^ T cells [12, 29]. Although IL-33 did not induce that differentiation [30], it polarized naïve CD4^+^ T cells into atypical Th2 cells that produced IL-5 and IL-13, but not IL-4 [31]. These results suggest that IL-25 and IL-33 play some roles in induction of typical or atypical Th2 cells in the sensitization phase that results in exacerbation of OVA-induced eosinophilic airway inflammation in the challenge phase. To elucidate this issue, we cultured DLN such as inguinal and axillary lymph nodes and spleen cells from EC OVA-sensitized WT mice, IL-25^-/-^ mice and IL-33^-/-^ mice in the presence and absence of OVA. As a result, the levels of Th2 and Th17 cytokines in the culture supernatants of DLN and spleen cells from IL-25^-/-^ mice and IL-33^-/-^ mice were comparable to those for WT mice (Fig 2A–2D and S3, S4, S5, S6 Tables). The levels of IL-25, IL-33 and TSLP in the culture supernatants of all groups were below the limit of detection by ELISA (data not shown). These results indicate that IL-25 and IL-33 are not essential for OVA-specific T-cell expansion in the sensitization phase at skin sites, at least in this setting.

### IL-25 and IL-33 are required for development of eosinophilic—but not neutrophilic—airway inflammation

**Fig 3:**
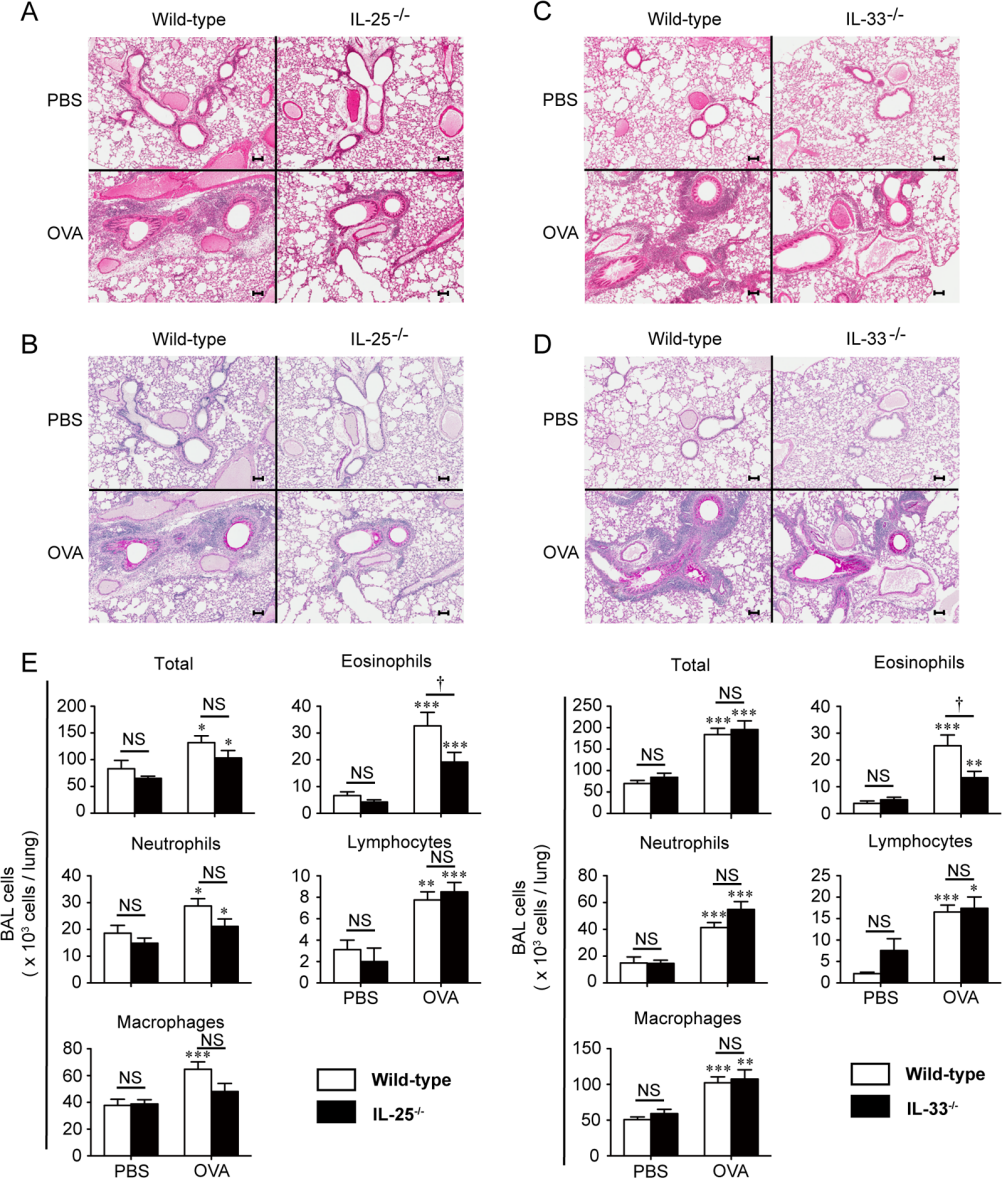
IL-25 and IL-33 are crucial for development of airway eosinophilia, but not neutrophilia, after the last intranasal OVA challenge in mice sensitized epicutaneously (EC) with OVA. Mice were sensitized EC with OVA three times and then treated intranasally with OVA or PBS for 3 consecutive days. Twenty-four hours after the last inhalation of OVA or PBS, BALFs and lungs were collected. (A, B) Lung sections from wild-type (WT) and IL-25^-/-^ mice were stained with hematoxylin-eosin (H-E) (A) and PAS (B). (C, D) Lung sections from WT and IL-33^-/-^ mice were stained with H-E (C) and PAS (D). Scale bars = 100 mm. The data show representative results from 10–14 mice in each experimental group, as indicated. (E) The numbers of BAL cells from WT (PBS, n = 8; OVA, n = 24) and IL-25^-/-^ mice (PBS, n = 8; OVA, n = 28), and from WT (PBS, n = 10; OVA, n = 20) and IL-33^-/-^ mice (PBS, n = 11; OVA, n = 20). Data are pooled from three independent experiments, each of which gave similar results. Data show the mean + SEM (E, F). *P < 0.05, **P < 0.01 and ***P < 0.001 vs. the corresponding values for PBS-treated mice. †*P* < 0.05 and NS (not significant) vs. the indicated group (WT vs. IL-25^-/-^, or WT vs. IL-33^-/-^).

Allergic airway inflammation accompanied by eosinophil and neutrophil infiltration, goblet cell hyperplasia and mucus secretion was observed in the lungs of EC OVA-sensitized WT mice after OVA challenge (Fig 3A–3D). On the other hand, infiltration of inflammatory cells was attenuated in the lungs of IL-25^-/-^ mice and IL-33^-/-^ mice in comparison with the WT mice. However, goblet cell hyperplasia and mucus secretion in the lungs of IL-25^-/-^ mice and IL-33^-/-^ were comparable to in the WT mice (Fig 3A–3D).

**Fig 4:**
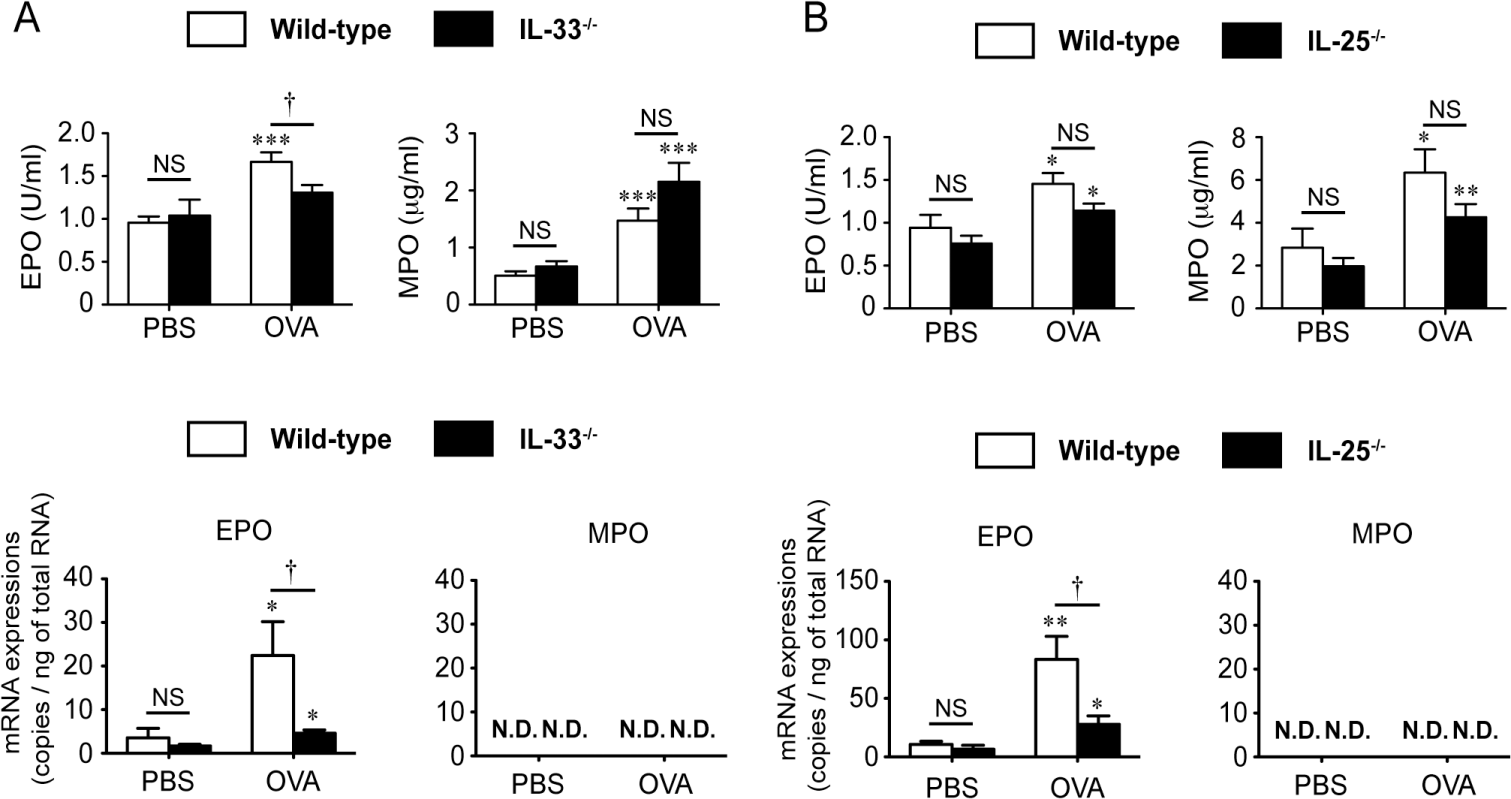
Contributions of IL-25 and IL-33 to eosinophil recruitment in lungs. The enzymatic activity and mRNA expression levels of EPO and MPO in BAL fluids and lungs, respectively, from wild-type (WT), IL-25^-/-^ mice and IL-33^-/-^ mice shown in Fig 3. Data are pooled from three independent experiments, each of which gave similar results. Data show the mean + SEM. *P < 0.05, **P < 0.01 and ***P < 0.001 vs. the corresponding values for PBS-treated mice. †*P* < 0.05 and NS (not significant) vs. the indicated group (WT vs. IL-25^-/-^, or WT vs. IL-33^-/-^).

Consistent with our earlier observations [17], the numbers of such cells as eosinophils, neutrophils, macrophages and lymphocytes in BALFs were significantly increased in EC OVA-sensitized WT mice after intranasal OVA challenge in comparison with after PBS challenge (Fig 3E and S7 Table). On the other hand, the eosinophil counts, but not the total-cell, neutrophil, macrophage or lymphocyte counts, in BALFs from IL-25^-/-^ mice and IL-33^-/-^ mice were significantly decreased compared with the WT mice (Fig 3E and S7 and S8 Tables). In association with the eosinophil counts in the BALFs, the levels of EPO mRNA in the lungs and EPO activity in BALFs were significantly decreased in IL-33^-/-^ mice compared with WT mice (Fig 4A and S9 Table). The results in IL-25^-/-^ mice vs. WT mice were similar to those in IL-33^-/-^ mice: the levels of EPO mRNA in lungs were significantly decreased, while the levels of EPO activity in the BALFs were slightly, but not significantly, decreased (Fig 4B and S10 Table). Moreover, consistent with the neutrophil and macrophage counts, the levels of MPO activity in BALFs from IL-25^-/-^ mice and IL-33^-/-^ mice were comparable to those in the WT mice (Fig 4A and 4B, and S9 and S10 Tables). The expression of MPO mRNA was below the limit of detection with qPCR in all groups (Fig 4A and 4B, and S9 and S10 Tables). These observations suggest that, in this setting, both IL-25 and IL-33 are responsible for induction of airway eosinophilia in local lesions in the challenge phase, rather than OVA-specific T-cell expansion in the sensitization phase.

### IL-25 and IL-33 are required for Th2-type, but not Th17-type, immune responses in mice after EC antigen sensitization

**Fig 5:**
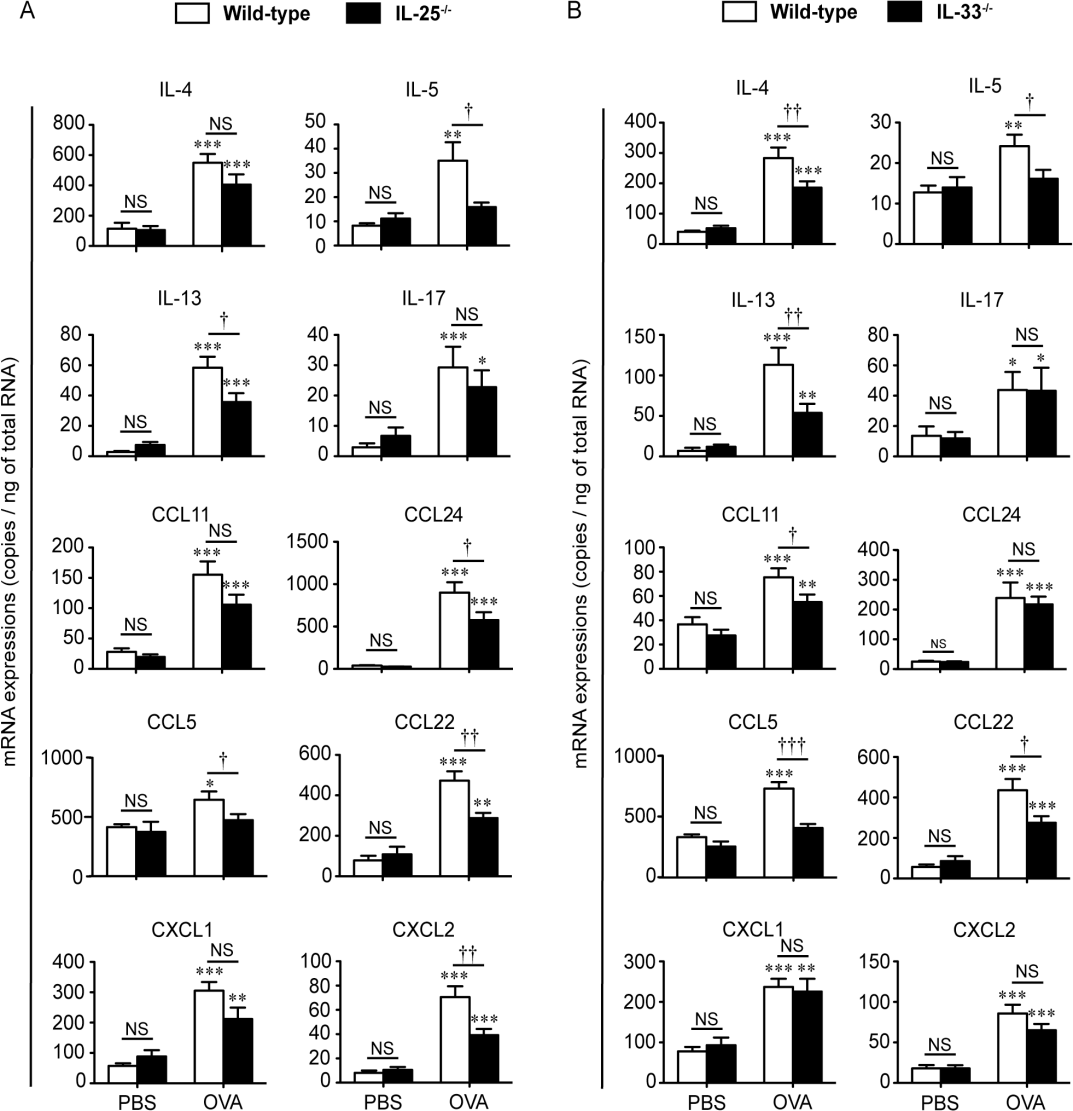
IL-25 and IL-33 are important for expression of Th2-type/associated cytokines and chemokines, but not Th17-type cytokines, after the last intranasal OVA challenge, in lungs from mice sensitized epicutaneously (EC) with OVA. Total mRNA was isolated from the lungs of wild-type (WT), IL-25^-/-^ mice and IL-33^-/-^ mice as shown in Fig 2E. The expression of mRNA for cytokines and chemokines was determined by quantitative PCR. (A) WT (PBS, n = 8; OVA, n = 24) and IL-25^-/-^ mice (PBS, n = 8; OVA, n = 28). (B) WT (PBS, n = 10; OVA, n = 20) and IL-33^-/-^ mice (PBS, n = 11; OVA, n = 20). Data are pooled from three independent experiments, each of which gave similar results. Data show the mean + SEM. *P < 0.05, **P < 0.01 and ***P < 0.001 vs. the corresponding values for PBS-treated mice. †*P* < 0.05, ††*P* < 0.01 and NS (not significant) vs. the indicated group (WT vs. IL-25^-/-^, or WT vs. IL-33^-/-^).

As we reported previously [17], expression of mRNA for Th2 cytokines (i.e., IL-4, IL-5 and IL-13), Th2 associated chemokines (i.e., CCL5, CCL11, CCL22 and CCL24), IL-17A and neutrophil chemoattractants (i.e., CXCL1 and CXCL2) was increased in the lungs of EC OVA-sensitized WT mice after intranasal OVA challenge in comparison with after intranasal PBS challenge (Fig 5A and 5B and S11 and S12 Tables). Consistent with the eosinophil count and EPO activity, mRNA expression for IL-5, IL-13, CCL5, CCL22 and CCL24, but not IL-4 or CCL11, in the lungs was significantly decreased after intranasal OVA challenge of IL-25^-/-^ mice compared with the WT mice (Fig 5A and S11 Table). Decreased mRNA expression was seen for IL-4, IL-5, IL-13, CCL11, CCL5 and CCL22, but not CCL24, in the lungs of IL-33^-/-^ mice (Fig 5B and S12 Table). On the other hand, in association with the neutrophil count and MPO activity, mRNA expression for IL-17A and CXCL1, but not CXCL2, in the lungs of IL-25^-/-^ mice was comparable with in the WT mice after intranasal OVA challenge (Fig 5A and S11 Table). Nearly similar results were obtained for mRNA expression for IL-17A, CXCL1 and CXCL2 in the lungs of IL-33^-/-^ mice (Fig 5B and S12 Table). Although we examined the protein levels of cytokines tested as in Fig 5 by ELISA, these cytokines were below the limit of detection of ELISA (data not shown).

### IL-25 and IL-33 are not required for antigen-induced airway hyperresponsiveness in EC sensitized mice

**Fig 6:**
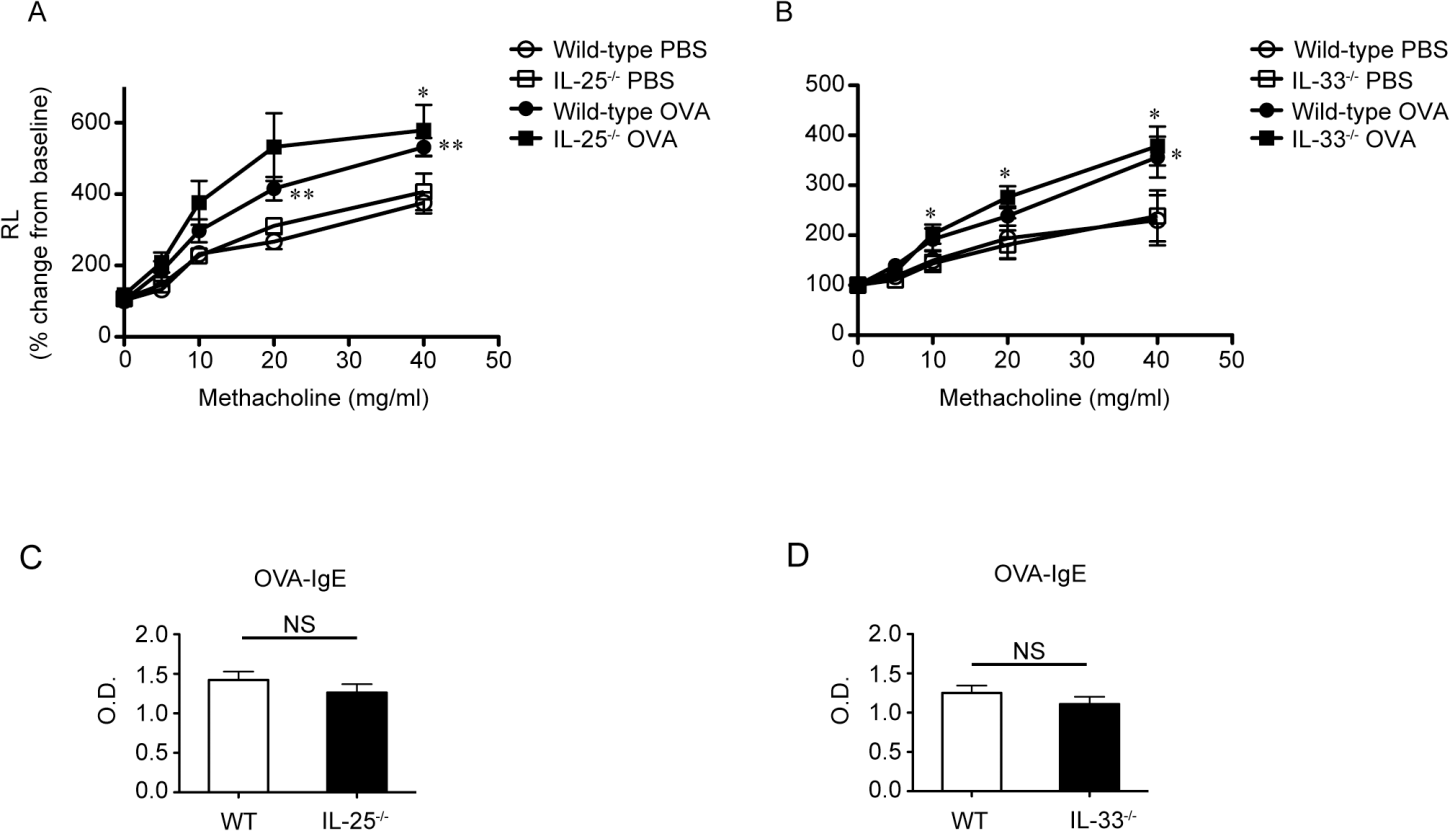
IL-25 and IL-33 are not required for airway hyperresponsiveness after the last intranasal OVA challenge in mice sensitized epicutaneously (EC) with OVA. Twenty-four hours after the last inhalation of OVA or PBS, mice sensitized EC with OVA were monitored for pulmonary resistance in response to methacholine. (A) Wild-type (WT) (PBS, n = 7; OVA, n = 7) and IL-25^-/-^ mice (PBS, n = 7; OVA, n = 7). (B) WT (PBS, n = 5; OVA, n = 8) and IL-33^-/-^ mice (PBS, n = 5; OVA, n = 12). (C, D) The levels of OVA-specific IgE in sera from mice after intranasal challenge were determined by ELISA. Data show the mean + SEM. *P < 0.05 and **P < 0.01 vs. the corresponding values for PBS-treated mice (A, B). NS (not significant) vs. the indicated group (WT vs. IL-25^-/-^, or WT vs. IL-33^-/-^)(C, D).

As previously reported [32], airway hyperresponsiveness (AHR) to methacholine in WT mice was significantly enhanced after intranasal OVA challenge compared with after intranasal PBS challenge (Fig 6A and 6B and S13 and S14 Tables). Although the eosinophil counts in BALFs from IL-25^-/-^ mice and IL-33^-/-^ mice were significantly decreased compared with WT mice after intranasal OVA challenge (Fig 3E, and S7 and S8 Tables), AHR to methacholine after intranasal OVA challenge was comparable in all three mouse strains (Fig 6A and 6B, and S13 and S14 Tables). In addition, the OVA-specific IgE levels in sera from IL-25^-/-^ mice and IL-33^-/-^ mice were comparable to those in WT mice (Fig 6C and 6D and S15 and S16 Tables). These observations indicate that neither IL-25 nor IL-33 is essential for induction of either AHR or antigen-specific IgE production in the setting.

## Discussion

We and other investigators had reported that IL-25, IL-33 and TSLP were crucial for development of airway inflammation in mice sensitized “intraperitoneally” with OVA emulsified with alum [18, 23, 33]. However, IL-33, but not IL-25 or TSLP, was shown to be responsible for development of airway inflammation in mice sensitized “intranasally” with house dust mite antigens [24]. That is, IL-33 induced innate lymphoid cell expansion and enhanced OX40L expression on dendritic cells, resulting in activation of antigen-specific Th2 cells [24]. On the other hand, TSLP, which is induced in skin by tape-stripping, is known to be important for development of airway inflammation in mice sensitized “epicutaneously” with OVA (in the absence of alum) by promoting Langerhans cell activation [34, 35]. These observations suggest that the molecular mechanisms underlying the pathogenesis of allergic airway inflammation [28] differ depending on the route of antigen sensitization (i.e., intraperitoneal, intranasal or EC sensitization) and the kind of antigen [26].

As noted above, TSLP is important for development of airway inflammation in mice sensitized EC with OVA after tape-stripping [34, 35]. We also previously demonstrated that development of airway inflammation was significantly attenuated in ST2^-/-^ mice sensitized EC with OVA after tape-stripping [17], suggesting that the IL-33-IL-33R (ST2) pathway is responsible for development of eosinophilic airway inflammation in EC antigen-sensitized mice. However, in certain mouse models for diseases such as allergic airway inflammation induced by intraperitoneal immunization with OVA [10, 18, 19], collagen-induced arthritis [20, 21] and LPS-induced septic shock [18, 36], distinctly different immune responses were observed between ST2^-/-^ mice and IL-33^-/-^ mice. Those earlier findings suggest that IL-33^-/-^ mice, rather than ST2^-/-^ mice, should be used to elucidate the role of IL-33 in development of eosinophilic airway inflammation in EC antigen-sensitized mice. In addition to IL-33, it has been unclear whether IL-25 is involved in airway inflammation in mice sensitized EC with OVA after tape-stripping. In the present study, we clearly demonstrated that airway eosinophilia was significantly reduced in EC sensitized IL-25^-/-^ mice and EC sensitized IL-33^-/-^ mice compared with EC sensitized WT mice (Fig 3E and S7 and S8 Tables), indicating that both IL-25 and IL-33 are crucial for development of airway inflammation in the setting.

TSLP is known to be important for dendritic cell migration from the skin to draining LNs and dendritic cell activation, following Th2 cell activation [34, 35, 37]. On the other hand, neither IL-33 nor IL-25 was essential for that migration [18] (and data not shown). Like TSLP [38, 39], both IL-25 and IL-33 can promote Th2 cell activation [6, 7]. On the other hand, we previously demonstrated that neither IL-25 nor IL-33 is crucial for OVA-specific T-cell induction and activation in the sensitization phase of airway inflammation in mice sensitized intraperitoneally with OVA/alum [18, 23]. Likewise, in the present study, we found that OVA-specific T-cell induction and activation in DLN and spleen cells from IL-25^-/-^ mice and IL-33^-/-^ mice after EC sensitization with OVA were similar to in WT mice (Fig 2A–2D and S3, S4, S5, S6 Tables). Although constitutive expression of IL-25 mRNA and increased expression of IL-33 mRNA after tape-stripping in the skin were observed (Fig 1A and S1 Table), neither skin-derived IL-25 nor IL-33 is essential for induction and activation of skin dendritic cells or OVA-specific T cells in the sensitization phase of airway inflammation in mice sensitized EC with OVA. Thus, these observations suggest that, in the setting, lung epithelial cell-derived, rather than skin-derived, IL-25 and IL-33 are important for induction of development of airway inflammation by activating immune cells such as Th2 cells, mast cells, eosinophils and/or innate lymphoid cells in the local lesion in the challenge phase, rather than in the sensitization phase.

Airway inflammation in mice sensitized intraperitoneally with OVA/alum is known to be mediated predominantly by Th2-type immune responses [40]. On the other hand, airway inflammation in mice sensitized EC with OVA was characterized by accumulation of not only eosinophils but also neutrophils, and airway eosinophilia and neutrophilia were mediated by Th2-type and Th17-type immune responses, respectively [4]. In the present study, we found that the number of eosinophils, but not neutrophils, was reduced in the BALFs from EC sensitized IL-25^-/-^ mice and EC sensitized IL-33^-/-^ mice compared with EC-sensitized WT mice after challenge with OVA (Fig 3E and S7 and S8 Tables). Consistent with this, the mRNA expression levels of Th2-type cytokines (IL-4, IL-5 and/or IL-13), but not IL-17, in the lungs of EC sensitized IL-25^-/-^ mice and EC sensitized IL-33^-/-^ mice were also reduced compared with EC sensitized WT mice after the last intranasal challenge with OVA (Fig 5A and 5B and S11 and S12 Tables). On the other hand, although AHR to methacholine was attenuated in both IL-25^-/-^ mice and IL-33^-/-^ mice sensitized intraperitoneally with OVA/alum [18, 23], we found here that it was comparable in both EC sensitized IL-25^-/-^ mice (Fig 6A and S13 Table) and EC sensitized IL-33^-/-^ mice (Fig 6B and S14 Table) to in EC sensitized WT mice after the last intranasal OVA challenge. These results indicate that both IL-25 and IL-33 are critical for induction of Th2-type cytokine-mediated allergic airway eosinophilia, but not Th17-type cytokine-mediated airway neutrophilia or AHR, in mice sensitized EC with OVA.

As noted above, different immune responses were observed between ST2^-/-^ mice and IL-33^-/-^ mice in allergic airway inflammation induced by “intraperitoneal” immunization of OVA [10, 18, 19], collagen-induced arthritis [20, 21] and LPS-induced septic shock [18, 36]. We also found some differences between EC sensitized ST2^-/-^ mice and EC sensitized IL-33^-/-^ mice in development of airway inflammation after OVA challenge. Compared with EC sensitized WT mice, the number of eosinophils in the BALF was significantly reduced in both EC sensitized ST2^-/-^ mice [17] and EC sensitized IL-33^-/-^ mice (Fig 3E, and S7 and S8 Tables) after OVA challenge. On the other hand, the number of macrophages in the BALFs was significantly decreased in EC sensitized ST2^-/-^ mice [17], but not EC sensitized IL-33^-/-^ mice (Fig 3E, and S7 and S8 Tables), after OVA challenge. In addition, EC sensitized ST2^-/-^ mice [17], but not EC sensitized IL-33^-/-^ mice (Figs 4A, 5B and 6D and S9, S12 and S16 tables), showed reduced levels of CXCL1 and CXCL2 mRNA in the lungs, MPO activity in the BALFs and OVA-specific IgE in the sera after OVA challenge. These observations suggest that ST2 may play an IL-33-independent role in development of eosinophilic airway inflammation in epicutaneously antigen-sensitized mice.

In summary, we demonstrated that both IL-25 and IL-33 are critical for induction of Th2-type cytokine-mediated allergic airway eosinophilia, but not Th17-type cytokine-mediated airway neutrophilia, at local lung sites in the challenge phase of mice sensitized EC with OVA, without affecting OVA-specific T-cell induction in the sensitization phase. However, neither IL-25 nor IL-33 was essential for induction of AHR to methacholine after the last intranasal OVA challenge in mice sensitized EC with OVA. Our findings provide new insight for development of novel treatments for asthma.

## Supporting Information

S1 Table Individual data depicted in Fig 1A. (XLSX)

S2 Table Individual data depicted in Fig 1B. (XLSX)

S3 Table Individual data depicted in Fig 2A. (XLSX)

S4 Table Individual data depicted in Fig 2B. (XLSX)

S5 Table Individual data depicted in Fig 2C. (XLSX)

S6 Table Individual data depicted in Fig 2D. (XLSX)

S7 Table Individual data depicted in Fig 3E (IL-25 KO). (XLSX)

S8 Table Individual data depicted in Fig 3E (IL-33 KO). (XLSX)

S9 Table Individual data depicted in Fig 4A. (XLSX)

S10 Table Individual data depicted in Fig 4B. (XLSX)

S11 Table Individual data depicted in Fig 5A. (XLSX)

S12 Table Individual data depicted in Fig 5B. (XLSX)

S13 Table Individual data depicted in Fig 6A. (XLSX)

S14 Table Individual data depicted in Fig 6B. (XLSX)

S15 Table Individual data depicted in Fig 6C. (XLSX)

S16 Table Individual data depicted in Fig 6D. (XLSX)
